# Does Intergenerational Care Increase Sugar-Sweetened Beverage Consumption of Schoolchildren? Evidence from CEPS Data in China

**DOI:** 10.3390/nu17142267

**Published:** 2025-07-09

**Authors:** Manjing Feng, Qi Liu, Dekun Du, Yanjun Ren

**Affiliations:** College of Economics & Management, Northwest A&F University, Yangling 712100, China; fengmanjing@nwafu.edu.cn (M.F.); qi.liu@nwafu.edu.cn (Q.L.); du.dekun@outlook.com (D.D.)

**Keywords:** intergenerational care, sugar-sweetened beverages, consumption, China

## Abstract

**Background/Objectives**: Intergenerational care plays a significant role in shaping household dietary quality and human capital development in China. Influenced by the legacy of the one-child policy, the care provided in these families often prioritizes child-focused practices. This study examines how intergenerational care influences schoolchildren’s sugar-sweetened beverage (SSB) consumption. **Methods**: This study utilizes data from the 2014–2015 China Education Panel Survey (CEPS) to investigate the impact of intergenerational care on schoolchildren’s dietary behaviors, with a focus on sugar-sweetened beverage (SSB) consumption. We apply both ordinary least squares (OLS) regression and the ordered logit model to estimate the impacts, and we use the instrumental variables approach to address potential endogeneity. **Results**: Schoolchildren from only-child families report greater SSB consumption, while those from multi-child families consume less. Intergenerational care is linked to more digital media exposure, more pocket money, and less parental supervision. These findings withstand rigorous validation through multiple robustness checks, including sample restriction strategies and propensity score matching (PSM) analysis. The effect is especially pronounced among boys, schoolchildren from families with higher parental education levels, and schoolchildren attending schools without formal nutrition education programs. **Conclusions**: The result indicates that intergenerational care significantly increases SSB consumption among schoolchildren from only-child families. Community nutrition and school health education programs can reduce schoolchildren’s SSB consumption, thereby lowering risks of obesity and other public health concerns.

## 1. Introduction

Childhood overweight and obesity have emerged as a critical public health concern during dietary transitions worldwide, and the consumption of sugar-sweetened beverages (SSBs) is identified as a pivotal etiological factor [1]. A growing body of research establishes excessive sugar intake as the primary determinant of nutrition-related health risks [2,3], and sugar-sweetened beverages contribute to weight by either stimulating appetite or suppressing satiety signaling [4]. The epidemiological data reveal that global childhood obesity rates reached 159 million cases in 2022, representing a threefold increase since 1990 [5]. China faces particularly severe challenges in this regard, as the Global Burden of Disease Study ranks it as the country with the highest absolute number of pediatric obesity cases [6]. Notably, Chinese boys and girls rank 70th and 102nd, respectively, in global obesity prevalence [5], underscoring the urgency of addressing this public health issue. A sugar-sweetened beverage is any drink with caloric sweeteners, such as carbonated soft drinks, sports drinks, energy drinks, fruit drinks, chocolate (or otherwise sweetened) milk, and sweetened coffee and tea [7]. The global sugar consumption has tripled over the past five decades [8], and the consumption rates of sugar-sweetened milk beverages (30%), and regular SSBs (25%) among Chinese children and adolescents substantially exceed those of adults [9]. Multi-regional investigations confirm this trajectory, including nationally representative surveys [10], South China studies [11], Northeast China analyses [12], and North China research [13]. Importantly, SSB consumption patterns in childhood exhibit longitudinal persistence and often extend into adolescence [14]. Given that adolescence constitutes a critical period for dietary habit formation [15], it is imperative to target sugar-reduction interventions for this demographic [16]. Consequently, the systematic investigation of the determinants and mechanisms underlying pediatric SSB consumption is essential for developing evidence-based strategies to reduce intake and advance the realization of the “Healthy China 2030” initiative.

Schoolchildren’s sugar-sweetened beverage consumption is influenced by various factors, with the family microsystem playing a pivotal role. Existing research primarily examines the influence of familial factors on children’s SSB consumption across three interrelated dimensions. First, the material environment significantly affects consumption feasibility. Household economic capital [17] and the accessibility of SSBs [18] increase children’s access to these beverages. Moreover, media exposure indirectly influences dietary behavior by shaping nutritional cognition [19]. Second, from a normative behavioral perspective, parental nutrition literacy contributes to the intergenerational transmission of implicit dietary norms [20,21]. In addition, explicit parental interventions, such as dietary restrictions, have been shown to directly affect adolescents’ beverage choices [22,23]. Third, the modeling effect represents a key mechanism of dietary transmission. Parents’ consumption of unhealthy foods is positively associated with children’s SSB intake [24], whereas health-supportive parental behaviors are inversely related to children’s SSB consumption [25]. Importantly, inadequate caregiving may further exacerbate environmental risks, particularly during early childhood. For example, maternal absence due to migration has been identified as a critical factor with potentially long-term adverse effects on a child’s development [26].

With the transformation of economic and social structures, the change in family structure has led to an increasing reliance on intergenerational care among schoolchildren in developing countries [27]. By 2015, 244 million individuals, roughly 3% of the global population, were involved in international migration [28]. By 2020, 22% of children in China (66.9 million) were in situations where their parents had moved within the country [29]. With the migration of both parents for employment, grandparents increasingly serve as primary caregivers for children [26,30,31], exerting substantial influences on children’s cognitive development and health-related well-being. Notably, the increasing involvement of grandparents in childcare is not limited to China but also encompasses countries such as the United States [32], Italy [33], and other European nations [34]. However, compared with Western countries, grandparents in China are more likely to take care of children [27,35]. Wu and Zhang (2017) [36] found that about 71% of children lived with their grandparents in the sample. In terms of cognitive development, grandparents’ care contributes to children’s social skills [37], but it may negatively affect adolescents’ non-cognitive skills [38]. Regarding health and well-being, grandparents’ support contributes to adolescents’ life satisfaction [39], yet it is also linked to declines in their grandchildren’s academic performance [31]. Moreover, grandparent care significantly increases the risk of childhood obesity [40], and it is detrimental to the health of rural children [41]. Intergenerational care often relaxes behavioral restrictions for children. Concurrently, the proliferation of social media increases screen time exposure among children, thereby elevating sedentary behavior and raising the likelihood of unhealthy food purchases [42].

It is predominantly qualitative rather than quantitative research that examines the impact of intergenerational care on schoolchildren’s SSB consumption [43]. In China, the accelerated advancement of industrialization and urbanization has significantly intensified interregional population mobility, leading to a large-scale demographic transition [44]. This transition has contributed to a distinct intergenerational division of family care responsibilities, whereby intergenerational care has evolved into a crucial institutional mechanism for reconciling the temporal–spatial conflicts between parental labor participation and child-rearing obligations [37]. Qualitative studies have identified both beneficial and detrimental dietary influences associated with grandparental care. On the one hand, grandparents may model healthy eating, encourage the consumption of nutritious foods, and provide children with greater autonomy in dietary decisions [42,45]. On the other hand, they often fail to restrict energy-dense, nutrient-poor foods. Some studies report that grandparents offer unhealthy foods, including SSBs, as rewards, tokens of affection, or means of pacification [40,46,47]. Other studies suggest that grandparents are moderate in providing unhealthy foods such as SSBs [43,48], and may even foster a healthy food environment by limiting access to unhealthy options [49]. It is more common to provide healthy foods and beverages for grandchildren than SSBs [50]. In conclusion, grandparent care plays an important role in shaping the nutritional environment and eating behavior of children and adolescents [39,47,51]. However, it is unclear whether and how intergenerational care affects schoolchildren’s consumption of SSBs in China.

Based on data from the 2014–2015 China Education Panel Survey (CEPS), this study systematically examines the impact of intergenerational care on schoolchildren’s consumption of SSBs, as well as the mechanisms underlying this relationship. To address potential endogeneity, this study uses the proportion of intergenerational care in the same region, excluding the current sample as an instrumental variable. Furthermore, this study identifies key mediating factors, including schoolchildren’s social media exposure time, TV media exposure time, pocket money, and parental supervision, to explore the main transmission channels through which intergenerational care influences schoolchildren’s consumption of SSBs.

The main contribution of this paper lies in three key aspects. Firstly, this study enriches the understanding of how the family environment influences schoolchildren’s dietary and consumption behavior. These results highlight the need for targeted interventions to regulate SSB consumption within these specific subgroups. Secondly, this study systematically examines the impact of intergenerational care on SSB consumption among middle school students from one-child families. This study contributes to the understanding of the underlying mechanisms through which grandparental care influences SSB consumption among junior high school students in only-child families. Lastly, this study establishes a framework of nutrition and health policy grounded in optimizing family environments. Through analysis of parent–child interaction dynamics, this study provides actionable strategies to cultivate healthy dietary habits in youth. Further, its empirical framework offers insights for developing countries to mitigate children’s unhealthy consumption practices. The remainder of this paper is organized as follows. Section 2 introduces the data sources, variable specifications, and research methodology. Section 3 reports the empirical findings. Section 4 presents a discussion. Section 5 offers a general conclusion.

## 2. Materials and Methods

### 2.1. Data and Variables

The data used in this study were selected from the 2014–2015 China Education Panel Survey (CEPS), a nationally representative dataset. The CEPS is a large-scale survey project conducted by the National Survey Research Center (NSRC) at Renmin University of China. The survey covers 112 schools and 438 classes nationwide. The CEPS dataset contains 20,000 students in 28 counties (districts) across the country. The CEPS dataset includes multiple levels, such as family, school, community, and macro social structure.

The dependent variable in this study is the frequency of schoolchildren’s SSB consumption, measured by the question “Do you often drink sugar-sweetened beverages (such as bubble tea) or carbonated drinks?” Schoolchildren’s options include “never, rarely, sometimes, often, always.”

As for the explanatory variables, intergenerational care is measured by the question “Who is the family member directly responsible for taking care of the child’s daily life (grandparents)?” [42].

The mechanism variable “social media exposure” is operationalized using the survey item, “On weekends, how many hours do you typically spend on internet use daily?” Given participants’ interval-based responses (e.g., 1–2 h, 3–4 h), midpoint values are calculated to quantify daily exposure duration. The “television media exposure” variable is measured analogously. The “pocket money” variable is assessed through the parental questionnaire item “How much pocket money does your child receive weekly?” “Parental supervision” is derived from caregiver-reported composite scores across six behavioral domains, including academic performance (exams/homework), conduct regulation at school, friendship, attire, internet usage, and television viewing.

As for the covariates, this study collects data on participants’ personal characteristics and family characteristics [13]. Data at the individual level include gender, age, ethnicity, whether children are overweight, whether they have been boarding, and whether they have attended health education classes in primary and secondary school (in Table 1). Data at the household level include parental education level, whether parents go out, family economic conditions, whether families use tap water, and whether families have flush toilets (in Table 1).

### 2.2. Econometric Model

To explore the impact of intergenerational care on schoolchildren’s consumption of SSBs, we follow the methodology used in previous studies [52,53] and use both OLS regression and ordered logit regression (Ologit). The baseline estimation model is as follows.

(1) $${beverage}_{it}=\alpha_{0t}+\alpha_{1}{care}_{it}+\gamma_{1}X_{it}+\varepsilon_{it}$$

where ${beverage}_{it}$ represents the consumption of SSBs by schoolchildren, and ${care}_{it}$ represents intergenerational care, equal to 1 if the schoolchildren are provided daily care by a grandparent or maternal grandparent and equal to 0 otherwise. $X_{it}$ is the control variable, including individual-level characteristics and family-level characteristics. In terms of individual characteristics, the control variables include gender, age, minority nationality, health education exposure, boarding status, and BMI. Regarding family characteristics, the control variables consist of parents’ education level, parents’ migrant employment status, family socioeconomic status, and household sanitation conditions. $\varepsilon_{it}$ is the random error term.

## 3. Results

### 3.1. The Effect of Intergenerational Care on Schoolchildren’s Consumption of SSBs

In Table 2, the regression results of the overall sample are shown in column (1) and column (2), the regression results of the sample of one-child families are shown in column (3) and column (4), and we show the regression results of the sample of multi-child families in column (5) and column (6). The results reveal that the effect of intergenerational care on schoolchildren’s consumption of SSBs is not uniformly significant across all groups. Specifically, intergenerational care does not exert a significant influence on SSB consumption among schoolchildren from multi-child families. However, a significant and positive association is observed among schoolchildren from one-child families. For the one-child families, the estimates from both OLS and Ologit models consistently demonstrate significant and positive coefficients for intergenerational care, indicating a certain level of robustness in the findings. Moreover, the coefficients of the control variables are also reasonable. Mothers’ education is negatively correlated with schoolchildren’s consumption of SSBs [54], boys consume SSBs more frequently than girls, and family economic conditions are positively correlated with schoolchildren’s consumption of SSBs. This means that mothers with higher education levels show greater concern about their children’s unhealthy diets, while schoolchildren from a wealthy family exhibit stronger susceptibility to SSBs.

### 3.2. Robustness Checks and Endogeneity Analysis

#### 3.2.1. Impact of Intergenerational Care by Using PSM

To address potential estimation bias arising from self-selection, this study employs the propensity score matching (PSM) method. Firstly, the families with intergenerational care are treated as the treatment group, and the families without intergenerational care are treated as the control group, with the control variables taken as covariates. Secondly, samples of the control group are selected according to a variety of matching methods, including 3-nearest-neighbor matching, kernel matching, radius matching, and local linear regression matching. Finally, the average treatment effects on the treated (ATT) of intergenerational care is estimated. The results, presented in Table 3, indicate that intergenerational care has no significant effect on schoolchildren’s consumption of SSBs for the overall sample or multi-child families. However, for one-child families, the ATT is significant and positive. This further demonstrates that the results of this study are robust.

#### 3.2.2. Excluding Households Without Co-Residing Grandparents

To confirm that the positive effect on schoolchildren’s SSB consumption is attributed to grandparent care rather than parental care, the sample is limited to households living with grandparents. The regression results using OLS and ordered logit (Ologit) models are shown in Table 4. For one-child families, the coefficient of intergenerational care is still significant and positive. The robustness of the above analysis results is further confirmed.

#### 3.2.3. Endogeneity Analysis Results

To address the endogeneity problem caused by potentially omitted variables and reverse causality, this study employs an instrumental variable (IV) approach to validate the robustness of the primary findings. The IV is the proportion of intergenerational care in the same area excluding the current sample. The OLS model and the ordered logit model are used to estimate the impact of intergenerational care on schoolchildren’s consumption of SSBs. However, in terms of technical feasibility, the instrumental variable method cannot be directly applied to the ranking model. For binary selection models containing endogenous variables, the IV–Oprobit model can be used for estimation. Therefore, in order to overcome the endogeneity problem of intergenerational care, this study uses the conditional mixed process (CMP) estimation method for regression analysis. Following Roodman (2011) [55], the combination of IV and CMP estimation can better solve the endogeneity problem. The results in Table 5 show that the regression results of the IV–2SLS model are consistent with those of the CMP–Oprobit model. For families with only one child, the regression coefficient of intergenerational care and schoolchildren’s consumption of SSBs is significant and positive, indicating that there is a causal relationship between intergenerational care and schoolchildren’s consumption of SSBs in families with only one child. We cannot confirm the causal relationship for multi-child families. This proves the robustness of the results of this study.

### 3.3. Mechanism Analysis

To investigate the mechanisms linking intergenerational care to SSBs among schoolchildren in one-child households, we conduct regression analyses that incorporated digital media exposure, television exposure, pocket money, and parental supervision as mediating variables. In Table 6, column (1) shows that intergenerational care significantly increases schoolchildren’s digital media exposure. Column (3) reveals a negative association between intergenerational care and parental supervision, while column (5) indicates a positive correlation with more pocket money. Collectively, these results suggest that intergenerational care amplifies schoolchildren’s SSB consumption through three pathways, increasing digital media exposure, increasing pocket money, and reducing parental supervision. By contrast, traditional television media exhibits no significant effect, likely due to schoolchildren’s preferences for social media platforms over conventional TV for exposure to dietary marketing [56].

### 3.4. Heterogeneity Analysis Results

#### 3.4.1. Heterogeneity Analysis by Schoolchildren’s Gender

To investigate gender differences in the impact of intergenerational care on SSB consumption among schoolchildren from one-child families, this study conducts a heterogeneity analysis based on male and female samples. The results are shown in Table 7. For boys, the coefficient associated with intergenerational care is 0.088, which is significant at the 10% level. This indicates that there is a significant and positive correlation between intergenerational care and SSB consumption among boys. For girls, the results show no significant association. This finding suggests that the effect of intergenerational care on schoolchildren’s consumption of SSBs is more pronounced among boys. One possible reason is that boys may lack self-control and are more likely to develop unhealthy dietary habits.

#### 3.4.2. Heterogeneity Analysis by Parental Education Levels

To confirm family differences in how intergenerational care affects schoolchildren’s SSB consumption, we define those with parents whose highest educational attainment is high school or above as the high-education group and define those with parents whose highest educational attainment is middle school or below as the low-education group. From the perspective of parents’ educational background, we analyze the heterogeneity in the influence of intergenerational care on schoolchildren’s SSB consumption. As shown in Table 8, intergenerational care is significantly and positively associated with SSB consumption in the high-education group, whereas no significant relationship is found in the low-education group. This suggests that intergenerational care is more likely to lead to excessive SSB consumption among schoolchildren of parents with higher education levels, indicating that parental education and intergenerational care may interact to reduce unhealthy eating behaviors in schoolchildren.

#### 3.4.3. Heterogeneity Analysis by the Status of Whether Schoolchildren Receive Health Education

To investigate the differential effects of intergenerational care on schoolchildren’s SSB consumption, we specifically examine the implementation of school-based health education programs. Children who receive health education during either primary or junior secondary school are classified into the intervention group, and others comprise the control group. As presented in Table 9, intergenerational care demonstrates a significant and positive association with SSB consumption, specifically in the subgroup without school health education exposure. The reason may be that schoolchildren who lack formal health education have inadequate nutritional knowledge and weaker health awareness. These findings underscore the necessity of leveraging institutional educational advantages. The implementation of evidence-based health education curricula can effectively enhance children’s nutritional literacy and self-regulation capacities, thereby mitigating excessive SSB consumption.

## 4. Discussion

This study examines the impact of intergenerational care on schoolchildren’s consumption of SSBs and explores the underlying transmission mechanisms. Using data from the 2014–2015 China Education Panel Survey (CEPS), we focus on grandparents’ care behavior rather than living with grandparents to more accurately measure intergenerational care indicators. In addition, to address potential endogeneity, we employ the proportion of intergenerational care in the same region (excluding the focal sample) as an instrumental variable. In order to ensure the robustness of the results, we apply the PSM method and conduct additional robustness checks by excluding samples not living with their grandparents. The results reveal that intergenerational care significantly increases SSB consumption among middle school children in one-child families.

These findings extend the literature on household environments and children’s consumption of sugar-sweetened beverages (SSBs) in developing countries. Existing research has established parental influences on children’s SSB intake, including parental education [54], parenting rules [18], parent modeling of food rules [25], and parental support [25]. Beyond these factors, intergenerational care emerges as another substantial determinant of children’s SSB consumption. Our findings provide an additional empirical support for this conceptual linkage.

For one-child families, intergenerational care increases schoolchildren’s consumption of SSBs because of the exposure to digital networks, pocket money, and parental supervision. Intergenerational care has been found to increase schoolchildren’s exposure to digital media, subsequently influencing their consumption of SSBs. Generally, grandparents find it challenging to supervise younger grandchildren alongside fulfilling their responsibilities, so they may rely on media as an accessible tool to keep children occupied [57]. Compared to television, digital devices are more difficult to regulate, as TV content adheres to scheduled time slots and is inherently easier to control [58]. When grandparents assume caregiving responsibilities, they exhibit greater permissiveness toward media usage, and grandfathers are more lenient than grandmothers [57]. This dynamic substantially elevates the risk of screen addiction among schoolchildren.

Marketing of SSBs is pervasive across television and digital media, significantly increasing the likelihood of children consuming SSBs [19,59]. Children and adolescents exhibit heightened vulnerability to food marketing influences due to factors including immature cognitive development, peer pressure, and excessive digital media exposure [13], which collectively drive increased consumption of SSBs. Greater screen time among children and adolescents is associated with the neglect of a healthy diet [60], and television can influence children’s consumption of SSBs by providing an environment that encourages frequent consumption of SSBs [61]. Celebrity involvement in food marketing further guides children’s SSB consumption behavior [62]. With the development of emerging technologies, television has increasingly been supplanted by smartphones, which promote youth access to the internet and social media platforms [63]. Marketing to younger consumers through digital media is more profitable and further increases youth exposure to unhealthy digital food and beverage promotions [56], leading to increased purchasing and consumption of SSBs.

Intergenerational care reduces opportunities for parental supervision and increases the likelihood of parental financial support, leading to increased consumption of SSBs among schoolchildren. First, in the intergenerational guardianship model, parents provide less direct supervision due to work mobility or living in different places, which weakens parents’ supervision, especially that of fathers [42]. Simultaneously, grandparents, influenced by traditional parenting concepts, often engage in “intergenerational spoiling.” Second, compensatory financial support becomes the key mediating variable, and parents compensate for the lack of companionship by increasing their financial allowances. However, due to limitations in nutritional cognition and self-control, children are prone to unhealthy consumption behaviors. Empirical evidence indicates that children who receive regular financial allowances consume approximately 12% more sugar from SSBs per week than those who do not [13]. Notably, intergenerational disparities in nutritional knowledge exacerbate this issue, as the grandparent generation exhibits substantially lower awareness of the health hazards associated with SSB consumption compared to the younger parent generation. Such intergenerational discrepancies can lead to excessive consumption of these beverages among schoolchildren in intergenerational care households, thereby significantly increasing the risks of obesity and dental caries.

Consistent with previous literature, our study confirms that increased intergenerational care is associated with greater screen time among children [42]. With the declining influence of TV media and the growing prominence of social media, children who use digital online media, impacted by self-regulation ability and peer pressure [13,63], are more likely to consume SSBs [64]. Similarly, intergenerational care weakens parental supervision [42], and the lack of parental companionship is compensated for by providing higher living allowances [65], further exacerbating SSB consumption among children. Children’s consumption of SSBs should be addressed through family nutrition and health education, along with the establishment of rules for time management and pocket money allocation.

The heterogeneity analysis shows that there exist multidimensional differences in the impact of intergenerational care on schoolchildren’s SSB consumption. Specifically, at the individual level, intergenerational care in one-child families is positive and significantly associated with SSB consumption in boys, whereas no such significant effect is observed among girls. It is possible that a majority of girls adhere to regulating their beverage intake, whereas boys may present more heterogeneity in beverage consumption [66]. At the family level, intergenerational care in families with higher parental education levels has a more prominent role in promoting schoolchildren’s consumption of SSBs. At the school level, intergenerational care significantly increases schoolchildren’s SSB consumption if children do not have health education in schools. This means that educational intervention yields a modest effect on reducing SSB consumption [67]. This finding underscores the need to establish a tripartite intervention framework between individual, familial, and institutional dimensions, and the finding focuses on potential risks to children’s nutritional health stemming from intergenerational care.

This study acknowledges several methodological limitations that warrant consideration. First, the lack of quantitative data on schoolchildren’s exact SSB intake amounts means that we have to rely solely on consumption frequency metrics, which may compromise measurement accuracy. Furthermore, the mechanistic exploration remains incomplete due to inherent complexities in mapping the causal pathways through which intergenerational care influences schoolchildren’s dietary behaviors. While multiple plausible transmission mechanisms exist (e.g., caregiver purchasing patterns, intergenerational health literacy transmission, and family meal dynamics), the current analytical framework, constrained by data availability and variable operationalization limitations, focuses on verifying several predominant transmission pathways rather than exhaustively examining all potential mediators.

## 5. Conclusions

Conclusively, intergenerational care significantly increases SSB consumption among schoolchildren from only-child families. While the intergenerational care paradigm remains a deeply embedded element of multigenerational household structures in contemporary China, it is associated with heightened diet-related health risks, particularly the enhancement of SSB consumption among schoolchildren. Therefore, it is essential to establish targeted policy measures. Based on empirical findings, this study proposes three evidence-based interventions to mitigate excessive SSB intake. First, policies should focus on grandparents who are involved in childcare, improve their health literacy through channels such as community nutrition classes, and focus on improving parenting behaviors such as SSB selection and screen time management. Second, it is necessary to guide parents to recognize the mechanism of digital media addiction and reduce schoolchildren’s screen exposure time by limiting the usage of digital devices and implementing other intervention measures. Parents should enhance their supervisory role in schoolchildren’s daily routines, improve parent–child communication through digital tools, and foster children’s self-regulatory skills and dietary habits. Finally, it is necessary to integrate comprehensive nutrition and health education into school curricula.

## Figures and Tables

**Table 1:** Descriptive statistics.

Variables	Define	Observations	Mean	Std. Dev.
Beverage	Frequency of consumption of sugary or carbonated beverages	7294	2.834	0.857
Care	Whether they are cared for by grandparents	7294	0.228	0.420
Gender	Male/female	7294	0.505	0.500
Age		7294	13.901	0.858
EM	Ethnic Minority or not	7294	0.086	0.281
Healedu	Whether health education is taught in primary and secondary schools	7294	0.776	0.417
Lodge	Whether boarding at school	7294	0.300	0.458
BMI	Overweight or not	7294	0.084	0.278
Moedu	Mother’s education level	7294	2.262	1.036
Faedu	Father’s education level	7294	2.430	0.981
Faworkout	Father working outside the home	7294	0.143	0.350
Maworkout	Mother working outside the home	7294	0.104	0.305
Economic	The income level of the family	7294	2.952	0.598
Tapwater	Whether the home has running water	7294	0.903	0.296
Flushtoilet	Whether the home has a flush toilet	7294	0.795	0.404

**Table 2:** Estimated effects of intergenerational care on schoolchildren’s consumption of SSBs.

	All Families		One-Child Families		Multi-Child Families	
Variables	OLS	Ologit	OLS	Ologit	OLS	Ologit
Care	0.033	0.065	0.078 **	0.160 **	−0.009	−0.022
	(0.025)	(0.055)	(0.037)	(0.081)	(0.034)	(0.076)
Gender	0.092 ***	0.202 ***	0.064 **	0.117 *	0.102 ***	0.246 ***
	(0.020)	(0.044)	(0.030)	(0.067)	(0.027)	(0.061)
Age	−0.049 ***	−0.103 ***	−0.010	−0.015	−0.064 ***	−0.136 ***
	(0.016)	(0.035)	(0.027)	(0.059)	(0.020)	(0.045)
EM	0.089 *	0.182 *	0.020	0.033	0.162 **	0.324 **
	(0.046)	(0.100)	(0.066)	(0.145)	(0.065)	(0.139)
Healedu	−0.013	−0.021	−0.020	−0.035	−0.008	−0.016
	(0.013)	(0.029)	(0.020)	(0.044)	(0.017)	(0.038)
Lodge	0.048	0.113 *	0.090	0.194	0.051	0.124
	(0.031)	(0.068)	(0.058)	(0.128)	(0.037)	(0.083)
BMI	−0.074 **	−0.134 *	−0.068	−0.109	−0.074	−0.149
	(0.036)	(0.079)	(0.049)	(0.108)	(0.054)	(0.119)
Moedu	−0.019	−0.042	−0.043 **	−0.095 **	0.002	0.006
	(0.013)	(0.030)	(0.019)	(0.042)	(0.020)	(0.045)
Faedu	−0.015	−0.026	−0.028	−0.051	−0.005	−0.010
	(0.014)	(0.030)	(0.019)	(0.041)	(0.020)	(0.045)
Faworkout	0.046	0.109	0.106 **	0.248 **	−0.002	−0.005
	(0.036)	(0.080)	(0.054)	(0.119)	(0.049)	(0.107)
Moworkout	0.036	0.097	0.054	0.127	0.043	0.133
	(0.043)	(0.098)	(0.071)	(0.163)	(0.056)	(0.124)
Economic	0.076 ***	0.166 ***	0.080 **	0.160 **	0.074 ***	0.168 ***
	(0.020)	(0.046)	(0.033)	(0.073)	(0.027)	(0.060)
Tapwater	0.057	0.147 *	0.105	0.254	0.038	0.104
	(0.037)	(0.084)	(0.084)	(0.197)	(0.041)	(0.094)
Flushtoilet	0.107 ***	0.231 ***	0.040	0.049	0.121 ***	0.268 ***
	(0.032)	(0.071)	(0.061)	(0.140)	(0.037)	(0.084)
Constant	3.203 ***	-	2.834 ***	-	3.301 ***	-
	(0.241)	-	(0.395)	-	(0.310)	-
Cut1	-	−3.982 ***	-	−3.244 ***	-	−4.232 ***
	-	(0.538)	-	(0.873)	-	(0.704)
Cut2	-	−1.237 **	-	−0.452	-	−1.505 **
	-	(0.536)	-	(0.871)	-	(0.701)
Cut3	-	0.686	-	1.501 *	-	0.414
	-	(0.537)	-	(0.872)	-	(0.701)
Cut4	-	3.087 ***	-	3.797 ***	-	2.935 ***
	-	(0.542)	-	(0.876)	-	(0.710)
Regional fixed effect	Yes	Yes	Yes	Yes	Yes	Yes
Time fixed effect	Yes	Yes	Yes	Yes	Yes	Yes
Observations	7294	7294	3330	3330	3964	3964
R-squared	0.038	-	0.036	-	0.049	-

Note: standard errors in parentheses * *p* < 0.1, ** *p* < 0.05, *** *p* < 0.01.

**Table 3:** Results from PSM.

**All Families**	**3-Nearest Neighbor Matching**	**Kernel Matching**	**Radius Matching**	**Local Linear Regression Matching**
ATT	0.026	0.023	0.025	0.029
	(0.031)	(0.028)	(0.028)	(0.034)
Control variables	Yes	Yes	Yes	Yes
Observations	7294	7294	7294	7294
**One-Child Families**	**3-Nearest Neighbor Matching**	**Kernel Matching**	**Radius Matching**	**Local Linear Regression Matching**
ATT	0.090 **	0.090 **	0.078 **	0.097 **
	(0.043)	(0.039)	(0.040)	(0.046)
Control variables	Yes	Yes	Yes	Yes
Observations	3330	3330	3330	3330
**Multi-Child Families**	**3-Nearest Neighbor Matching**	**Kernel Matching**	**Radius Matching**	**Local Linear Regression Matching**
ATT	−0.026	−0.030	−0.026	−0.026
	(0.044)	(0.040)	(0.041)	(0.050)
Control variables	Yes	Yes	Yes	Yes
Observations	3964	3964	3964	3964

Note: Standard errors in parentheses * *p* < 0.1, ** *p* < 0.05, *** *p* < 0.01.

**Table 4:** Robustness test results by excluding households without co-residing grandparents.

Variables	All Families		One-Child Families		Multi-Child Families	
	OLS	Ologit	OLS	Ologit	OLS	Ologit
Care	0.054 *	0.096	0.109 ***	0.223 **	−0.003	−0.034
	(0.028)	(0.062)	(0.041)	(0.089)	(0.039)	(0.087)
Control variables	Yes	Yes	Yes	Yes	Yes	Yes
Regional fixed effect	Yes	Yes	Yes	Yes	Yes	Yes
Time fixed effect	Yes	Yes	Yes	Yes	Yes	Yes
Observations	6861	6861	3152	3152	3709	3709
R-squared/pseudo R-squared	0.037	0.015	0.035	0.014	0.049	0.021

Note: standard errors in parentheses * *p* < 0.1, ** *p* < 0.05, *** *p* < 0.01.

**Table 5:** Results from the endogeneity analysis.

	IV–2SLS			CMP–Oprobit		
Variables	All Families	One-Child Families	Multi-Child Families	All Families	One-Child Families	Multi-Child Families
Care	0.029	0.071 *	−0.010	0.035	0.087 *	−0.014
	(0.026)	(0.038)	(0.035)	(0.033)	(0.048)	(0.045)
Control variables	Yes	Yes	Yes	Yes	Yes	Yes
Regional fixed effect	Yes	Yes	Yes	Yes	Yes	Yes
Time fixed effect	Yes	Yes	Yes	Yes	Yes	Yes
Observations	7294	3330	3964	7294	3330	3964
R-squared	0.038	0.036	0.049	-	-	-

Note: standard errors in parentheses * *p* < 0.1, ** *p* < 0.05, *** *p* < 0.01.

**Table 6:** The results of the mechanism analysis.

Variables	Web Screen	Beverage	Parental Supervision	Beverage	Pocket Money	Beverage	TV Screen
Care	0.302 ***		−0.171 *		0.105 *		0.146
	(0.110)		(0.103)		(0.060)		(0.104)
Web screen		0.080 ***					
		(0.006)					
Parental supervision				−0.040 ***			
				(0.007)			
Pocket money						0.100 ***	
						(0.011)	
Control variables	Yes	Yes	Yes	Yes	Yes	Yes	Yes
Regional fixed effect	Yes	Yes	Yes	Yes	Yes	Yes	Yes
Time fixed effect	Yes	Yes	Yes	Yes	Yes	Yes	Yes
Observations	3330	3330	3330	3330	3330	3330	3330
R-squared	0.092	0.090	0.052	0.047	0.092	0.061	0.069

Note: standard errors in parentheses * *p* < 0.1, ** *p* < 0.05, *** *p* < 0.01.

**Table 7:** Results of heterogeneity analysis by schoolchildren’s gender.

Variables	Female	Male
Care	0.073	0.088 *
	(0.052)	(0.052)
Control variables	Yes	Yes
Regional fixed effect	Yes	Yes
Time fixed effect	Yes	Yes
Observations	1538	1792
R-squared	0.062	0.038

Note: standard errors in parentheses * *p* < 0.1, ** *p* < 0.05, *** *p* < 0.01.

**Table 8:** Results of heterogeneity analysis by parental education levels.

Variables	Low Education Group	High Education Group
Care	0.042	0.092 **
	(0.060)	(0.047)
Control variables	Yes	Yes
Regional fixed effect	Yes	Yes
Time fixed effect	Yes	Yes
Observations	1294	2036
R-squared	0.068	0.043

Note: standard errors in parentheses * *p* < 0.1, ** *p* < 0.05, *** *p* < 0.01.

**Table 9:** Results of heterogeneity analysis by whether schoolchildren receive health education.

Variables	Without Health Education	With Health Education
Care	0.168 *	0.056
	(0.091)	(0.040)
Control variables	Yes	Yes
Regional fixed effect	Yes	Yes
Time fixed effect	Yes	Yes
Observations	662	2668
R-squared	0.056	0.046

Note: standard errors in parentheses * *p* < 0.1, ** *p* < 0.05, *** *p* < 0.01.

## Data Availability

The data presented in this study are available at http://ceps.ruc.edu.cn/.

## Abbreviations

The following abbreviations are used in this manuscript:

SSBs	Sugar-sweetened beverages
PSM	Propensity score matching
CEPS	China Education Panel Survey
IV	Instrumental variable
ATT	Average treatment effects on the treated
Ologit	Ordered logit
